# Educated for Nothing

**Published:** 1871-12

**Authors:** 


### EDUCATED FOR NOTHING.

At the New York University, in the city of New York, under the presidency of Chancellor Crosby, a complete classical education may be obtained by any young man of good moral character without any charge whatever for tuition. How would that little boy's heart have almost jumped out of his body, who offered a schoolmaster to chop his wood, make his fires, do anything by which he could make himself useful, if in return he would teach him a little while every day, and which little boy subsequently became Daniel Webster.

Board can be had in New York, including washing, for ten dollars a week, and for several dollars less in some places. We know a gentleman, a man of education and distinction, who gets a good room in a good looking house not four blocks from Fifth Avenue, for two dollars a week. He extemporizes a breakfast for ten cents, and gets a good hearty dinner of meat, bread and butter, and one vegetable for twenty, and a light supper for eight. A young man thus living would have no incurable dyspepsia before he had passed half his course, and having good health, would stand the best chance of graduating at the head of his class.

